# A de novo Reciprocal X; 9 Translocation in A Patient with Premature Ovarian Failure

**Published:** 2013-07-31

**Authors:** Mir Davood Omrani, Soraya Saleh Gargari, Faezeh Azizi

**Affiliations:** 1 Department of Medical Genetics, Faculty of Medicine, Shahid Beheshti University of Medical Sciences, Tehran, Iran; 2 Obstetrics and Gynecology Unit, Mahdyeh Hospital, Shahid Beh eshti University of Medical Sciences, Tehran, Iran

**Keywords:** Premature Ovarian Failure, Translocation, Amenorrhea

## Abstract

Premature ovarian failure (POF) causes hypergonadotrophic amenorrhea in 1-3% of females, occurring before the age of 40 among women with chromosomal rearrangements in the long arm of the X chromosome 'critical region'. In this article, we report a case of POF and primary amenorrheain a girl with a de novo reciprocal translocation between chromosomes X and 9. The proband was a 17 years old girl with a history of irregular menstruation and high level of follicle-stimulating hormone (FSH) (151 mlU/mL) and luteinizing hormone (LH) (56 mlU/mL). In ultrasound examination, left ovarian gonad was atrophic without any follicles. Right ovarian gonad was not seen. Cytogenetical analysis was performed on the patient and her parents. Her karyotype results was 46, X, rcp (X; 9) (q24; q13) dn. Her parents had normal karyotype. This reciprocal translocation between chromosome X and 9 and observed POF in the patient suggest either the disruption of a critical gene expression due to 'position effect' or deletion of one or more POF-related genes in the disrupted long arm of the affected X chromosome.

## Introduction

Premature ovarian failure (POF) is one of the major causes of hypergonadotrophic amenorrhea among females before the age of 40, with the frequency range of about 1-3% (1). This syndrome was first described among young women having menopausal levels of follicle stimulating hormone (FSH), low estrogen levels and amenorrhea (2). Most causes of POF cases are unknown (3), but in the remaining cases diverse etiologies have been reported including: genetic aberrations, autoimmune ovarian damage, iatrogenic factors, infectious agents, toxins and environmental factors (4). Chromosome anomalies are responsible for 6-8% of POF cases (1). Abnormal X chromosome was seen in the majority of the cases and its association with ovarian dysgenesis was suggested (5). POF-related abnormalities range from partial to complete absence of one X chromosome to mutations at the DNA level. The disease is genetically heterogeneous (6).

Although in many cases, X monosomy is the major chromosomal finding, but other chromosomal abnormalities such as X-chromosome rearrangements, containing inversions, X/autosomal balanced translocations has been also reported (7). On the X-chromosome, the majority of the breakpoints are concentrated on the long arm, spreading over about half of its length. This region has been called the "Xq critical region" since it is necessary for the ovarian function and normal reproductive function (8). These regions are located between Xq13 and Xq26 (9) containing two groups of candidate POF-related genes: POF1 within Xq26-q27 (10) and POF2 within Xq13-q21 (11). Heterochromatic Region 1 supposes to yield a position effect on autosomal genes in reciprocal balanced translocations. POF correlated to this region is perhaps due to unbalanced expression of one or more translocated autosomal genes. No known X linked genes exist with ovarian-specific expression in this region.

Region 2, in the distal part of long arm of chromosome X is a fairly gene-rich region. Ovary expressed genes in this region may be needed in a double dose for the function of ovary, and their alterations by mutations maybe responsible for POF. Disturbing either of these regions, could cause different symptoms (12, 13). In our country many people refer to infertility centers for treatment. Finding and reporting chromosomal anomalies of POF could help to understand and improve the knowledge on the genetic causes of their condition and family planning. The aim of the present study was exploring the new causes of infertility and helping to reach to new insights in this scope.

Cytogenetical analysis was carried out on the patient and her parents, according to standard cytogenetical procedures with some modification (14). From each sample a minimum of 50 metaphases was surveyed, by using the Applied Imaging CytoVision Karyotyping System (Santa Clara, CA).Informed written consent was obtained from the parents of the patient. This study was approved in the Ethics Committee of Shahid Beheshti University of Medical Sciences.

## Case Report

The proband was an Iranian 17 years-old girl of non-consanguineous parents with a history of irregular menstruation, every 7-8 months. No history of birth defects, mental retardation and congenital malformations was seen in the family. Pregnancy was normal and at birth the patient had no dysmorphic characteristics. In ultrasound examination, her uterine was normal but her left ovarian gonad was small and atrophic without any follicles. Right ovarian gonad was not seen. Her serum follicle-stimulating hormone (FSH) and luteinizing hormone (LH) levels were high, 151 mlU/mL and 56 mlU/mL respectively. Her height was 160 cm at the time of diagnosis.

**Fig 1:**
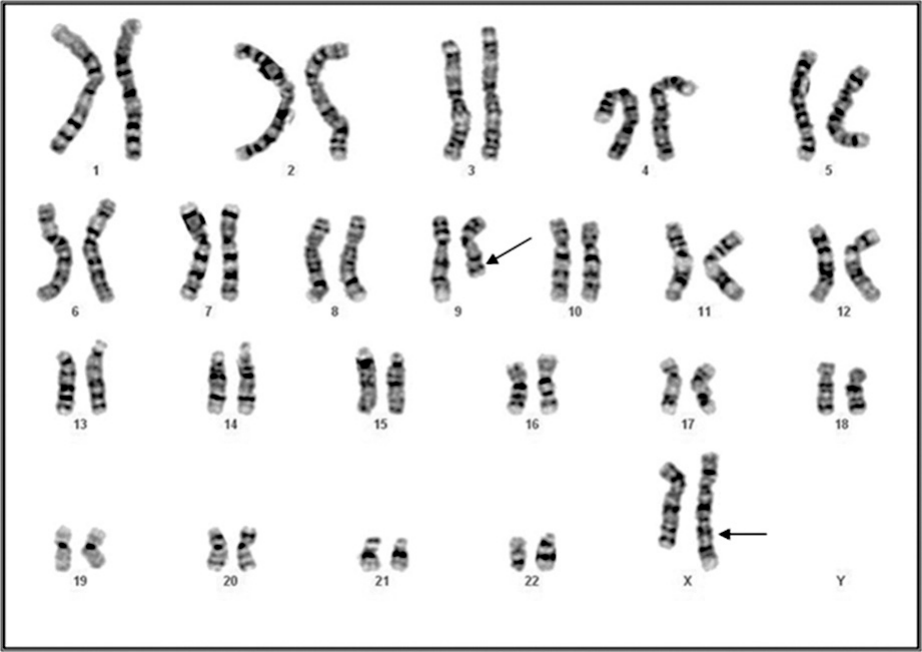
GTG banded chromosome typig in the daughter of the family showing reciprocal X; 9 translocation.

GTG banded karyotype of the patient showed a de novo reciprocal translocations between chromosomes X and 9. Her karyotype was 46, X, rcp (X; 9) (q24; q13)dn. No chromosomal abnormalities were noticed in her parents (Fig 1).

Cytogenetical analysis was carried out on the patient and her parents, according to standard cytogenetical procedures with some modification (14). From each sample a minimum of 50 metaphases was surveyed, by using the Applied Imaging CytoVision Karyotyping System (Santa Clara, CA). Informed written consent was obtained from the parents of the patient. This study was approved in the Ethics Committee of Shahid Beheshti University of Medical Sciences.

## Discussion

The pattern of X inactivation has been moderately unchanged and the normal X having been selectively inactivated in almost all cases. A normal phenotype would be anticipated in the existence of a balanced karyotype and seemingly normally functional X genes, even if they were divided into two translocated segments (15). De novo translocations between chromosome X and autosomal chromosomes are rare (16) but still chromosomal rearrangements are the major causes of POF (6). Balanced chromosomal translocation in the POFrelated genic regions could lead to the POF by several genetic mechanisms including reduced gene dosage, position effect and non-specific chromosome effects that impair eiosis. These can result in the failure of ovaries by decreasing the number of primordial follicles, increased atresia of the ovarian follicles due to apoptosis, or failure of follicle maturation (4, 17). Genes involved in balanced translocations signify new candidates for POF (12, 13). Deletions of the distal region of the POF1 locus are related with POF at ages 24- 39 years but POF at an earlier age of 16-21 years has been associated with translocations affecting the POF2 locus (18). Various translocations have been reported between X and other somatic chromosomes that lead to POF such as t (X; 17) (q22; q25) (19), 46, X, t (X; 15) (q24; q26.3) (20), 46, X, der (Y) t (X; Y)(q13.1; q11. 223) (1) and der (X) t (X; 11) (q28; p13) (21). Many of these translocations occur in critical regions of X chromosome which contain various genes necessary for ovarian normal reproductive function (1, 8) such as FMR1 (Xq27), FMR2(Xq28), DIAPH2 (Xq22), XPNPEP2 (Xq25), FSHPRH1 (Xq22) and some autosomal genes like, FSHR (2p21-p16), Inhibin A (2q33-q36), GALT (9p13) and NOGGIN (17q22) (18). Though, population-specific studies are still limited, occasionally a single mutation may prove significant in certain populations like FSHR in Finland and INH-A in Iran (4, 22). Here we report a case with irregular menstruation due to reciprocal translocation involving Xq24 and 9q13 regions. This breakpoint of the X chromosome was well inside the "critical region" of the X chromosome, therefore it is probable that her chromosome abnormality is responsible for her clinical state. A similar translocation at Xq22 region in a girl was reported who had delayed puberty and primary amenorrhea (16). In addition similar phenotype was reported in an 18-year-old girl with an X-autosome translocation t (X; 9) (q22; q12) (23).

Based on these findings including ours, it is recommended to carry out karyotyping as part of the basic evaluation of women diagnosed with POF due to repeated abnormal karyotypes (13-50%) in this situation. Having this information may aid and impact the family with their future decision making.
